# Generic amyloid fibrillation of TMEM106B in patient with Parkinson’s disease dementia and normal elders

**DOI:** 10.1038/s41422-022-00665-3

**Published:** 2022-04-27

**Authors:** Yun Fan, Qinyue Zhao, Wencheng Xia, Youqi Tao, Wenbo Yu, Mingjia Chen, Yiqi Liu, Jue Zhao, Yan Shen, Yunpeng Sun, Chenfang Si, Shenqing Zhang, Yaoyang Zhang, Wensheng Li, Cong Liu, Jian Wang, Dan Li

**Affiliations:** 1 Department of Neurology and National Research Center for Aging and Medicine & National Center for Neurological Disorders, State Key Laboratory of Medical Neurobiology, Huashan Hospital, Fudan University, Shanghai, China; 2 Bio-X Institutes, Key Laboratory for the Genetics of Developmental and Neuropsychiatric Disorders (Ministry of Education), Shanghai Jiao Tong University, Shanghai, China; 3 Bio-X-Renji Hospital Research Center, Renji Hospital, School of Medicine, Shanghai Jiao Tong University, Shanghai, China; 4 Interdisciplinary Research Center on Biology and Chemistry, Shanghai Institute of Organic Chemistry, Chinese Academy of Sciences, Shanghai, China; 5 University of Chinese Academy of Sciences, Beijing, China; 6 Department of Anatomy and Histoembryology, School of Basic Medical Sciences, State Key Laboratory of Medical Neurobiology and MOE Frontiers Center for Brain Science, Institutes of Brain Science, Fudan University, Shanghai, China; 7 Zhangjiang Institute for Advanced Study, Shanghai Jiao Tong University, Shanghai, China

**Keywords:** Cryoelectron microscopy, Ageing

Dear Editor,

Protein amyloid aggregation is a histological hallmark of neurodegenerative diseases (NDs).^1,2^ In synucleinopathies including Parkinson’s disease (PD), Parkinson’s disease dementia (PDD), dementia with Lewy body and multiple system atrophy, α-synuclein (α-syn) is commonly characterized to form amyloid aggregates presenting distinct pathological activities in diseased brains.^3,4^ Structural characterization of α-syn amyloid fibrils formed in different diseases could provide mechanistic understanding of heterogeneous α-syn pathologies.^5,6^

In this work, we originally sought to extract α-syn amyloid fibrils from the brain tissue of a patient with PDD, together with the brain tissues of two normal elderly individuals as controls, for structural study (Supplementary information, Table S1). Notably, although normal 2 is a normal control, we observed β-amyloid (Aβ) and Tau pathologies in the brain (Supplementary information, Fig. S1). The PDD patient was in the late stage of PD and manifested severe dementia symptom (Supplementary information, Table S2). Immunohistochemistry characterization confirmed severe pS129-α-syn pathology in the postmortem brain of this patient (Supplementary information, Fig. S1).

To extract amyloid fibrils from the donor brains, we adopted previously published protocols^5,7^ with modifications (Supplementary information, Fig. S2a). Negative staining transmission electron microscopic (NS-TEM) imaging showed that the P4 pellets of PDD patient were buried under many amorphous aggregates (Supplementary information, Fig. S2b). We thus incubated the P4 pellets with pronase for different times (15–240 min), and observed that pronase treatment gradually cleared the contaminant aggregates while leaving clean fibrillar aggregates. We next adopted the same protocol to handle the brain tissues of two normal elders. Unexpectedly, we also extracted numerous fibrillar aggregates from the brains of these two normal elderly controls (Supplementary information, Fig. S2c), which prompted us to suspect the identity of the fibrils extracted from the PDD tissue.

Despite of our concern about the identity of the extracted fibrils, we moved forward to determine their cryo-EM structures, by which we expected an answer. We thus collected cryo-EM micrographs for the fibril samples and picked fibrils for 2D classification (Supplementary information, Table S3). One dominant species was identified in the fibrils from two normal elders; two species were identified in the fibrils from PDD patient, in which the major species (87.4%) exhibits a nearly identical length of helical half pitch (203 nm) to that of the fibril from normal 1 (204 nm) (Fig. 1a). 3D reconstruction showed that fibrils from normals 1, 2 and the major species from PDD represent a single protofilament, while the minor species is composed of two protofilaments (Fig. 1a; Supplementary information, Fig. S3). Strikingly, cross-sections of the density maps revealed a new conformation that is unlike any known conformations of α-syn. Moreover, it appears that similar conformations were formed across the different fibrils.

**Fig. 1:**
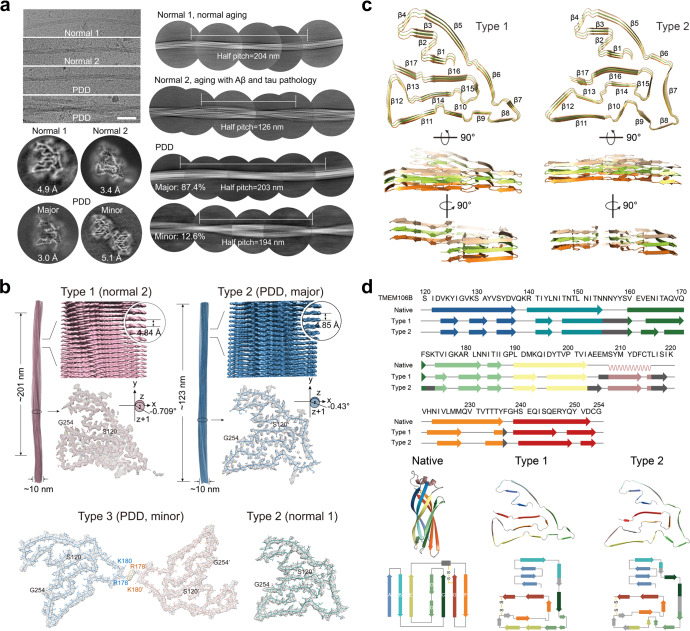
The cryo-EM structures of TMEM106B fibrils extracted from brains of PDD patient and normal elders. **a** Cryo-EM micrographs, 2D and 3D classification of amyloid fibrils extracted from the brains of two normal elders, and a PDD patient. The half pitch of the fibrils was determined by 1200-, 864-, 1024- and 1024-pixel box size class averages in 2D classification, respectively. Normal 2 has Aβ and Tau pathologies based on histological staining, while no clinical symptoms were manifested. Resolutions of 3D reconstruction are indicated. Scale bar, 50 μM. **b** Cryo-EM density maps and structural models (top view) of the Type 1 fibril extracted from normal 2 and Type 2 fibril extracted from PDD. Fibril width, length of half pitch, helical rise and twist angle are indicated. The twist angle is graphically illustrated. Graphing was performed with UCSF Chimera v1.13 (top). Overlay of the Type 2 structure with the density map of Type 3 fibril extracted from PDD via rigid-body fitting. The two protofilaments in Type 3 fibril are colored in blue and pink, respectively (bottom left). Overlay of the Type 2 structure with the density map of the fibril extracted from normal 1 (bottom right). The terminal residues are labeled. **c** Three-layer structure models of Type 1 and Type 2 TMEM106B fibrils are shown in cartoon and displayed in different views. β-strands are indicated. **d** Primary sequence of TMEM106B (residues 120–254) and the secondary structure alignment of the Type 1 and Type 2 fibrils with the native structure (predicted by AlphaFold2). β-strands are colored based on their counterparts in the native structure (top). Structures of TMEM106B in these three states are shown in the middle. The topology diagrams of TMEM106B in the three structures are shown at the bottom.

We next sought to build structural models for the brain-extracted fibrils. However, trials of threading the α-syn sequence into the density map failed. To identify whether the fibrils are composed of α-syn, we conducted immunogold labeling combined with NS-TEM with the use of α-syn antibody (Supplementary information, Fig. S4). The result showed that neither the fibrils from PDD patient nor those from normal elders were able to be labeled by immunogold. In addition, Aβ and Tau antibodies cannot label the fibrils, either (Supplementary information, Fig. S4). While we were struggling with the identification of the unknown fibrils, we noticed a preprint work, showing amyloid fibril structures formed by transmembrane protein 106B (TMEM106B) extracted from human brains.^8^ Their cryo-EM densities appear similar to ours. Indeed, tandem mass spectrometry detected three digested peptides specific to TMEM106B in our fibrils (Supplementary information, Fig. S5), which confirmed that the fibrils that we obtained from the three donors are formed by TMEM106B.

We then were able to unambiguously fit TMEM106B molecules into the cryo-EM density maps and build structural models for the fibrils from normal 2 (referred to as Type 1) and the major species of PDD (referred to as Type 2) (Fig. 1b). As for the fibrils from normal 1 and the minor species from PDD, we directly settle the Type 2 structure into their density maps via rigid-body fitting. The Type 2 TMEM106B structure fits well in both density maps, except that TMEM106B dimerizes in the minor fibril species of PDD (referred to as Type 3) via electrostatic interactions between R178, K180 and an unknown density (Fig. 1b). In total, we obtained three types of fibril structures (polymorphs) from four fibril species extracted from the brains of three donors: Type 1 fibril in normal 2, Type 2 in normal 1 and PDD, and Type 3 in PDD. TMEM106B conformations in Type 2 and Type 3 (dimer) are nearly identical, while that in Type 1 is moderately different from the other two.

The fibril cores of Type 1 and Type 2 both consist of residues 120–254 of TMEM106B forming 17 β-strands, which are arranged into a curling stone-like fold (Fig. 1c). Notably, the N-terminal end Ser120 is deeply buried inside the fibril core, which excludes the existence of undetermined additional residues. In the native state, TMEM106B is cleaved by lysosomal proteases, which leads to the formation of the N-terminal fragment and the luminal domain.^9^ Thus, the structure of TMEM106B in the fibrillar state indicates that prior to forming amyloid fibrils, the luminal domain is cleaved off at Arg119. Interestingly, in the native state, residues 120–254 form a β-sandwich Ig-fold consisting of 7 β-strands as predicted by AlphaFold (Fig. 1d). Secondary structural alignment showed that while transforming from the native to fibrillar states, the original long β-strands were generally partitioned into shorter fragments to curl into a serpentine conformation (Fig. 1d). Noteworthily, the disulfide bond formed between Cys214 and Cys253 remains unchanged in both states, which locks the C-terminus to the rest of the fold.

Residues 120–160 build the handle of the curling stone and are nearly identical in conformation in both Type 1 and Type 2 fibrils (Supplementary information, Fig. S6a). In the handle part, βA in native TMEM106B is divided into three β-strands, in which β1 forms steric zipper-like interaction with β16 attaching the handle to the stone, and K129 and D136 form a salt bridge stabilizing the bending between β2 and β3 (Supplementary information, Fig. S6b, c).

In contrast to the identical N-terminal handle part of the structure, the stone part of Type 1 and Type 2 exhibits conformational variations. The overall fold of Type 2 is rather flat, while the stone part of Type 1 swaps between neighboring rungs (Fig. 1c). The protein segment (residues 174–186) forms a hydrophobic hole in Type 2 (Supplementary information, Fig. S6d), in which unknown densities were observed in the fibrils from normal 1 and PDD (Fig. 1a). In contrast, no additional density was observed in the counterpart of the Type 1 fibril, where the protein segment forms tight intramolecular hydrophobic interactions (Supplementary information, Fig. S6d). Thus, the binding of chemicals probably induces the shift of the rest of C-terminal residues, while the topologies of the folds in both types remain similar (Fig. 1d).

Moreover, four large unidentified electron densities were identified in the fibril core and are adjacent to Asn145, Asn151, Asn164, and Asn183 (Supplementary information, Fig. S7). These four Asn residues are on the outer surface of the native structure and have been identified to be post-translationally modified by glycosylation, which are required for the transportation of TMEM106B from endoplasmic reticulum to late endosome/lysosome.^10^ Our cryo-EM data showed that these four Asn residues remain glycosylated and point outwards in the fibril structure of TMEM106B, which also contributes to the conformational selection of the fibrils.

The role of TMEM106B in NDs is controversial. Previous evidence for the association of TMEM106B with NDs was poor, despite that increased expression levels and missense mutation of TMEM106B have been found in the patients with frontotemporal lobar degeneration.^11,12^ Until lately, it was reported that TMEM106B forms amyloid fibrils in the brains of patients with various NDs.^13,14^ However, we found that TMEM106B forms amyloid fibrils not only in the diseased brain, but also in the brains of normal elders. Notably, one of the normal elders (101 yr) has Aβ and Tau pathologies in the brain, although no ND phenotype was manifested. Consistent with our finding, a recent preprint study (published online during the revision of our work) showed that TMEM106B forms amyloid fibrils in human brains in an age-dependent manner,^8^ although in another study, no fibrils were managed to obtain from aged brains.^14^ Therefore, a serious debate is whether TMEM106B fibrils are associated with ND pathology or not. We summarized the donor information from our work and the recent three papers of TMEM106B fibrils^8,13,14^ (Supplementary information, Fig. S8 and Table S4). Interestingly, we found that the age of donors with NDs is significantly younger than that of normal elders, regardless of the classification of the patients by familial and sporadic cases or by different types of diseases. This indicates the correlation of TMEM106B fibril formation with NDs. However, a causative role of TMEM106B fibrils in NDs is less likely, considering the generic TMEM106B fibrillation in normal elders.

In summary, we report that in our attempt to purify α-syn fibrils from the postmortem brain of a patient with PDD, we obtained fibrils formed by TMEM106B instead. Intriguingly, using the same protocol, we also obtained TMEM106B fibrils from two normal elders (71 yr and 101 yr). Moreover, cryo-EM structure determination of these ex vivo fibrils showed that they share similar structures, in which TMEM106B adopts a curling stone-like fold composed of residues 120–254 that originally form the luminal domain of TMEM106B in its native fold. Our work demonstrates that TMEM106B commonly forms amyloid fibrils in diseased and aged brains with no necessary consequence of NDs, which highlights the complicated relationship between protein amyloid formation and the pathogenesis of NDs.

## Supplementary information

Supplementary information

## Data Availability

Cryo-EM 3D density maps have been deposited in the Electron Microscopy Data Bank (EMDB) with entry codes: EMD-33054 for Type 1 TMEM106B fibril in normal 2, and EMD-33055 for Type 2 TMEM106B fibril in PDD. The corresponding structure models have been deposited in the Worldwide Protein Data Bank (wwPDB) with entry codes: 7X83 for Type 1 TMEM106B fibrils, and 7X84 for Type 2 TMEM106B fibrils. Additional data that support findings of this study will be available from the corresponding authors upon reasonable request.
