# Supplementary material for: Generic amyloid fibrillation of TMEM106B in patient with Parkinson’s disease dementia and normal elders
Source: Cell Res. 2022 Apr 27;32(6):585–8. doi: 10.1038/s41422-022-00665-3 (PMC9160068; doi:10.1038/s41422-022-00665-3)
Supplement: Supplementary file 1 — Supplementary information [file 41422_2022_665_MOESM1_ESM.pdf]

Supplementary information

Generic amyloid fibrillation of TMEM106B in patient with Parkinson's disease dementia and normal elders

Yun Fan[1¶], Qinyue Zhao[2,3¶], Wencheng Xia[4,5¶], Youqi Tao[2,3], Wenbo Yu[1], Mingjia Chen[1], Yiqi Liu[1], Jue Zhao[1], Yan Shen[1], Yunpeng Sun[4,5], Chenfang Si[4,5], Shenqing Zhang[2,3], Yaoyang Zhang[4,5], Wensheng Li[6], Cong Liu[4,5*], Jian Wang[1*], Dan Li[2,3,7*]

[1]Department of Neurology and National Research Center for Aging and Medicine & National Center for Neurological Disorders, State Key Laboratory of Medical Neurobiology, Huashan Hospital, Fudan University, Shanghai 200040, China

[2]Bio-X Institutes, Key Laboratory for the Genetics of Developmental and Neuropsychiatric Disorders (Ministry of Education), Shanghai Jiao Tong University, Shanghai, China.

[3]Bio-X-Renji Hospital Research Center, Renji Hospital, School of Medicine, Shanghai Jiao Tong University, Shanghai 200240, China.

[4]Interdisciplinary Research Center on Biology and Chemistry, Shanghai Institute of Organic Chemistry, Chinese Academy of Sciences, Shanghai, 201210, China.

[5]University of Chinese Academy of Sciences, Beijing 100049, China.

[6]Department of Anatomy and Histoembryology, School of Basic Medical Sciences, State Key Laboratory of Medical Neurobiology and MOE Frontiers Center for Brain Science, Institutes of Brain Science, Fudan University, Shanghai 200032, China

[7]Zhangjiang Institute for Advanced Study, Shanghai Jiao Tong University, Shanghai 200240, China.

¶These authors contributed equally: Yun Fan, Qinyue Zhao, Wencheng Xia.

*To whom correspondence should be addressed. E-mails: lidan2017@sjtu.edu.cn, wangjian_hs@fudan.edu.cn, and liulab@sioc.ac.cn.

Materials and Methods

Post-mortem human brain samples

The brain samples of the three donors were acquired from the Body Donation Station in Fudan University (Shanghai Red Cross Society). The clinical data of the PDD patients were from Huashan Parkinson’s Disease Patient Follow-up Cohort Database. Ethical approval of this study was granted by the Human Studies Institutional Review Board, Huashan Hospital, Fudan University. Informed consent was obtained from the donors and their next of kin. All procedures conducted in the study were in conformity with the ethical standards of Declaration of Helsinki.

Extraction of amyloid fibrils

The extraction of amyloid fibrils from post-mortal frozen brain tissues was performed as previous reported[1, 2] with some modifications. In brief, frozen brain tissues, temporal cortex in normal 1 and normal 2, frontal cortex in PDD, were manually homogenized for three times in buffer containing 10 mM Tris-HCl (pH 7.5), 0.8 M NaCl, 10% sucrose,1 mM EGTA, 0.1% sarkosyl, PMSF and cocktail. Homogenates were brought to 2% sarkosyl and incubated at 37°C for 1 h with manually shaking after every 15 min for several times, followed by centrifugation at 10,000 g for 10 min at 4°C. Then, the supernatants (S1) were subjected to ultracentrifugation at 100,000 g for 60 min at 4°C. The pellets (P2) after the ultracentrifugation were collected and resuspended with extraction buffer (1,000 μl/g) following with a low-speed centrifugation at 3,000 g for 5 min at 4 °C. The supernatants (S3) were diluted into 3-fold with buffer consisting of 50 mM Tris-HCl (pH 7.5), 0.15 M NaCl, 10% sucrose and 0.2% sarkosyl and centrifugated at 166,000 g for 1 h at 4°C. The sarkosyl-insoluble pellets (P4) were resuspended with 20 mM Tris-HCl (pH 7.4) and 50 mM NaCl (100 μl/g). For NS-TEM, immuno-EM and cryo-EM, the sarkosyl-insoluble pellets were incubated with pronase for 1-3 hour to digest the amorphous aggregates and remove the fuzzy coat surrounding the amyloid fibril. Approximately 0.5 g brain

tissues were used for NS-TEM and immune-EM studies, and 1 g brain tissue were used for cryo-EM study.

Negative staining transmission electron microscopy (NS-TEM)

5 μL of brain-extracted sample solution was loaded onto the glow-discharged 230 mesh carbon coated copper grids (Beijing Zhongjingkeyi Technology Co., Ltd.). After incubation of 45 s, the sample was removed by the filter paper, and the grid was washed with 5 μl ddH_2O and 5 μl 2% w/v uranyl acetate sequentially. Then another 5 μl 2% w/v uranyl acetate was applied to stain the sample for 45 s. The sample imaging was accomplished by a Tecnai T12 microscope (FEI Company) operated at 120 kV.

Immuno-gold negative-staining electron microscopy

5 μL of *in vitro* recombinant α-syn fibrils (10 μM), $Aβ_{42}$ fibrils (2 μM), tau fibrils (10 μM) and brain-extracted sample solution was loaded onto the glow-discharged 230 mesh carbon coated copper grids (Beijing Zhongjingkeyi Technology Co., Ltd.), respectively, and incubated for 2 min with remaining sample removed by filter paper. The grids were blocked with 0.1% BSA for 5-10 min at room temperature. Then, the brain-extracted samples and the different *in vitro* recombinant fibrils as positive controls were incubated with primary antibody, including anti-α-syn (ab51253, Abcam), anti-Aβ (8243S, CST), and anti-tau (13-6400, Invitrogen), at 1:50 for 2 h at room temperature, respectively. After washing once with PBS, the grids were incubated with immunogold-labeled secondary antibodies, including anti-Rabbit antibody labeled with 12 nm colloidal gold (1:100, 711-205-152, Jackson Immuno Research) for samples stained with anti-α-syn and anti-Aβ antibodies, and anti-Mouse antibody labeled with 6 nm colloidal gold (1:100, 115-195-146, Jackson Immuno Research) for samples stained with anti-tau antibody, for 1 h at room temperature. After sequentially washing with PBS, ddH_2O and 2% w/v uranyl acetate, the grids were stained with 2% w/v uranyl acetate for 45 s. Then, the excess buffer was removed with filter paper. The infrared lamp was used to

further dry the grids. The negative control group of the *in vitro* recombinant fibrils was treated under the same conditions as that of the positive control group but without incubation of primary antibodies. The TEM micrographs were collected by a Tecnai T12 microscope (FEI Company) operated at 120 kV.

Immunohistochemistry staining

Brain tissues were fixed with 4% paraformaldehyde for 3 days, followed by gradient dehydration and cryotome sectioning. Brain sections (30 μM) were subjected to 3% hydrogen peroxide solution to quench its endogenous peroxidases. After blocked with 5% BSA/PBS, 5% goat serum plus 0.5% Triton X-100, sections were incubated with a primary antibody against p-α-syn (1:1000, ab51253, Abcam), Aβ (1:1000, 803015, BioLegend) or tau (1:1000, MN1020, Invitrogen) for 12-16 h at 4°C. Then the sections were sequentially incubated with a biotin-conjugated secondary antibody (1:3000, BA-1000, Vector) for 2 h and avidin-biotin complex (1:1000, PK-6100, Vector) for 3 h at RT. After labelling using a DAB-peroxidase substrate (SK-4100, Vector), the sections were counterstained with hematoxylin.

Mass spectrometry

The brain-extracted fibril sample (the sarkosyl-insoluble pellets (P4)) was firstly dissolved in 2M urea, 1M β-mercaptoethanol, 2% sodium dodecyl sulfate and the SDS-PAGE loading dye, then was sonicated for 40 cycles (1 s on/off per cycle) under 20% amplitude by JY92-IIN sonicator. Then the sample was boiled at 100°C for 10 min and loaded on 4–20% Bis–Tris gels (GenScript), following by electrophoresis at 200 V for 30 minutes. The gels were stained by Coomassie brilliant blue. The bands between 25 kDa and 50 kDa was collected by a clean scalpel into cubes for following mass spectrometry analysis. Gel pieces were destained using ammonium bicarbonate/ACN (1:1, vol/vol) at 37°C overnight, following with the treatment of neat ACN at room temperature for 20-40 min. Then, 10 mM DTT and 55 mM IAA were sequentially applied to treat the gel pieces at 56°C and room temperature for 10 minutes. After washing with

neat ACN, the gel pieces were digested by trypsin for 12-16 h at 37°C. After removing the digestion buffer to another 1.5 ml microtube, the gel pieces were further treated with buffer containing 5% formic acid (FA) and ACN (0.5, vol/vol) for 30 minutes at 37 °C to extract the potential digested peptides in gels as much as possible. Mixed with digestion buffer, the extraction buffer and digestion buffer mixture were centrifugated at 4,000 rpm for 20 minutes. The resultant supernatants were carefully removed to another 1.5 ml microtube and dried down in a vacuum centrifuge. Then the samples were resuspended in 20 µl 0.1% FA and used for following LC–MS/MS analysis.

The peptide mixture was analyzed using an on-line EASY-nL-LC 1000 coupled with an Orbitrap Fusion mass spectrometer. The sample was loaded directly onto a 15-cm home-made capillary column (C18-AQ, 1.9 mm, Dr.Maisch, 100 mm I.D.). Mobile phase A consisted of 0.1% FA, 2% ACN and 98% H_2O and mobile phase B consisted of 0.1% FA, 2% H_2O and 98% ACN. A 120 min gradient (mobile phase B: 3% at 0 min, 8% at 5 min, 20% at 92 min, 30% at 113 min, 95% at 115 min, 90% at 120 min) was used at a static flow rate of 300 nl / min. The data were acquired in a data-dependent (top-20) mode. For MS1, the scan range was set to 300 - 1800 m/z at a resolution of 120,000. The AGC target was set as 500,000 with a maximum injection time of 50 ms. For MS2 scan in the orbitrap, the resolution was set to 30,000. The AGC target was set as 50,000 and the maximum injection time was set to 100 ms. MS/MS raw sepctra were processed using Mascot search engine (version 2.8.0). The SwissProt human protein sequence database containing 20,350 sequence entries (download data: April 21, 2020) was used for database search. The precursor mass tolerance was set to 10 ppm, and the fragment mass tolerance was 0.02 Da. A fixed carbamidomethyl modification on cysteine, variable modifications of methionine oxidation were set. Trypsin was used as the enzyme, and up to 3 missed cleavage site was allowed. PSMs were filtered to 1% false discovery rate using the target-decoy strategy.

Cryo-EM sample preparation and data collection

After treated with pronase, the aqueous solution containing TMEM106B fibrils was applied to glow-discharged holey copper Quantifoil grids (300 mesh, R2/1 for normal 1 and normal 2, R1.2/1.3 for PDD). Then, the cryo-EM grids were plunge-frozen in liquid ethane after blotting with filter paper using Vitrobot Mark IV (FEI, Thermo). Cryo-EM micrographs (40 frames per micrograph) were collected on Thermo Fisher Titan Krios G4 cryo transmission electron microscope, operated at 300 kV with a BioContinuu K3 direct detector (Gatan), using a GIF Quantum energy filter (Gatan) with a slit width of 20 eV to remove inelastically scattered electrons. Super-resolution movies were recorded at ×105,000 magnification with a pixel size 0.83 Å $pixel^{-1}$ and the total dose was ~55 e^{-} $Å^{2}$ with exposure time of 2 s. Automated cryo-EM data collection was performed by using EPU software (Thermo) with defocus values from -1.4 to -2.2 μm.

Image pre-processing and helical reconstruction

For image pre-processing, 40 movie frames per micrograph were corrected for beam induced motion, aligned, dose-weighted, and further binned with a physical pixel size of 0.83 Å using MotionCorr2[3]. The contrast transfer function was estimated from motion-corrected images by CTFFIND-4.1.8[4]. Helical reconstruction was performed in RELION 3.1[5].

(1) Normal 1 and PDD dataset

For Type 2 fibril in normal 1, 6,856 manually picked fibrils from 1,041 micrographs were individually extracted to segments with a box size of 360 pixels and an inter-box distance of 29.9 Å. Then re-extract the particle with 1024 box size and downscaled to 440 pixels. The reference-free two-dimensional classification steps with a decreasing in-plane angular sampling rate from 8° to 0.5° and a T=2 regularization parameter were performed to calculate the half pitches from the 2D class averages and discard the segments contributing suboptimal 2D class averages. The Gaussian cylinder was calculated and used

for initial model. The optimal segments were selected and an initial 3D model that was low-pass-filtered to 60 Å were further applied to perform 3D classifications (K=3) using the helical parameters calculated through the splicing of 2D class averages. The class of segments that yielded the clearest 3D reconstruction map was selected and subjected to additional rounds of 3D classifications (K=1) with local optimization of helical twist and rise. Then, 3D auto-refinements with optimization of helical twist and rise after reconstructions were carried out. The purified segments through the above 2D and 3D classification were re-extracted using a smaller box size of 686 pixels with downscaling to 320 box size. Then the best 3D class was used as the initial model for 3D classification (K=1) with the inter-box diameter of 36.5 Å. To further improve the resolution of 3D reconstruction maps, we performed Bayesian polishing and contrast transfer function (CTF) refinement, followed by 3D auto-refinement. Finally, the maps were sharpened with a soft-edge solvent mask using the standard "post-processing" program in RELION 3.1[5]. Overall resolution estimates were calculated based on the gold-standard 0.143 Fourier shell correlation (FSC) between the two independently refined half-maps. Local resolution was estimated using the Local resolution procedure in RELION 3.1 with the same mask and B-factor in post-processing[5].

For PDD, the 4,816 manually picked fibrils from 440 micrographs were extracted to segments with a box size of 288 pixels and an inter-box distance of 23.9 Å, and then processed in the same way as normal 1. Differently, after 2D classification in 1024-pixel box size with rescaling to 440 pixels, we find two classes of fibril polymorphs, Type 2 (87.4% of all particles) and Type 3 (12.6% of all particles). Particles from Type 3 were selected by 3D classification (K=3). Subsequently, the 3D classes were used as initial model for several rounds of 3D classifications (K=1) and 3D auto-refinements. For Type 2 in PDD, the helical reconstruction procedures are the same as it in normal 1.

(2) Normal 2 dataset

For Type 1 fibril in normal 2, the 5,278 manually picked fibrils from 989 micrographs were extracted to segments with a box size of 864 pixels and an inter-box distance of 71.7 Å. Reference-free 2D classification with T = 2 regularization parameter was performed with the gradually decreasing in-plane rotation angular sampling rate from 8° to 0.5°. The purified segments were used to construct the initial 3D model in “relion_helix_inimodel2d” program to be applied as a reference map[5]. The generated initial 3D model and segments were applied to perform 3D classification (K=3) with the helical twist and rise parameters from the splicing of 2D class averages. Local search of symmetry to optimize the helical twist and rise was carried out after separation of β-strands was shown to select the clearest class. 3D auto-refinements with local optimization of helical twist and rise were executed followed by contrast transfer function (CTF) refinement to improve the resolution of 3D reconstruction map. The final reconstruction map was sharpened in using “Post-processing” program with a soft-edge solvent mask.

Atomic model building

According to the density map after post-processing, the atomic model of Type 1 fibril was built *de novo*, then the atomic model of Type 2 fibril was built based on map and structure model of Type 1 fibril in COOT[6]. Then, both two models with three layers were generated in Chimera and refined by real-space refinement program in PHENIX[7]. There are more additional details in Table 3.

Supplementary Figures

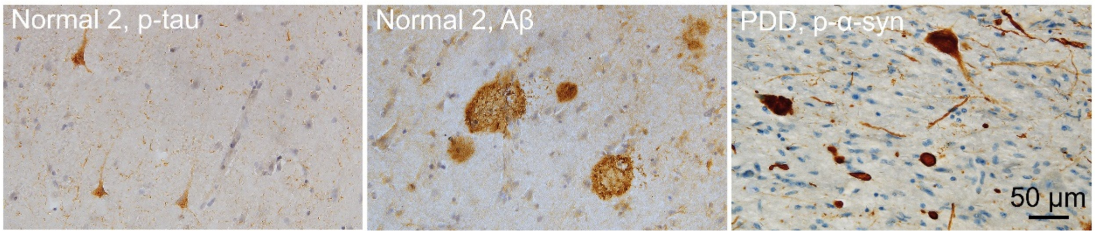

Fig. S1. Immunohistochemistry of the brain samples derived from normal 2 and PDD. Staining of p-tau inclusions (left), Aβ plaques (middle) in the temporal cortex of normal 2, and p-α-syn inclusions in the dorsal motor nucleus of vagus nerve of PDD (right). Scale bar, 50 μm.

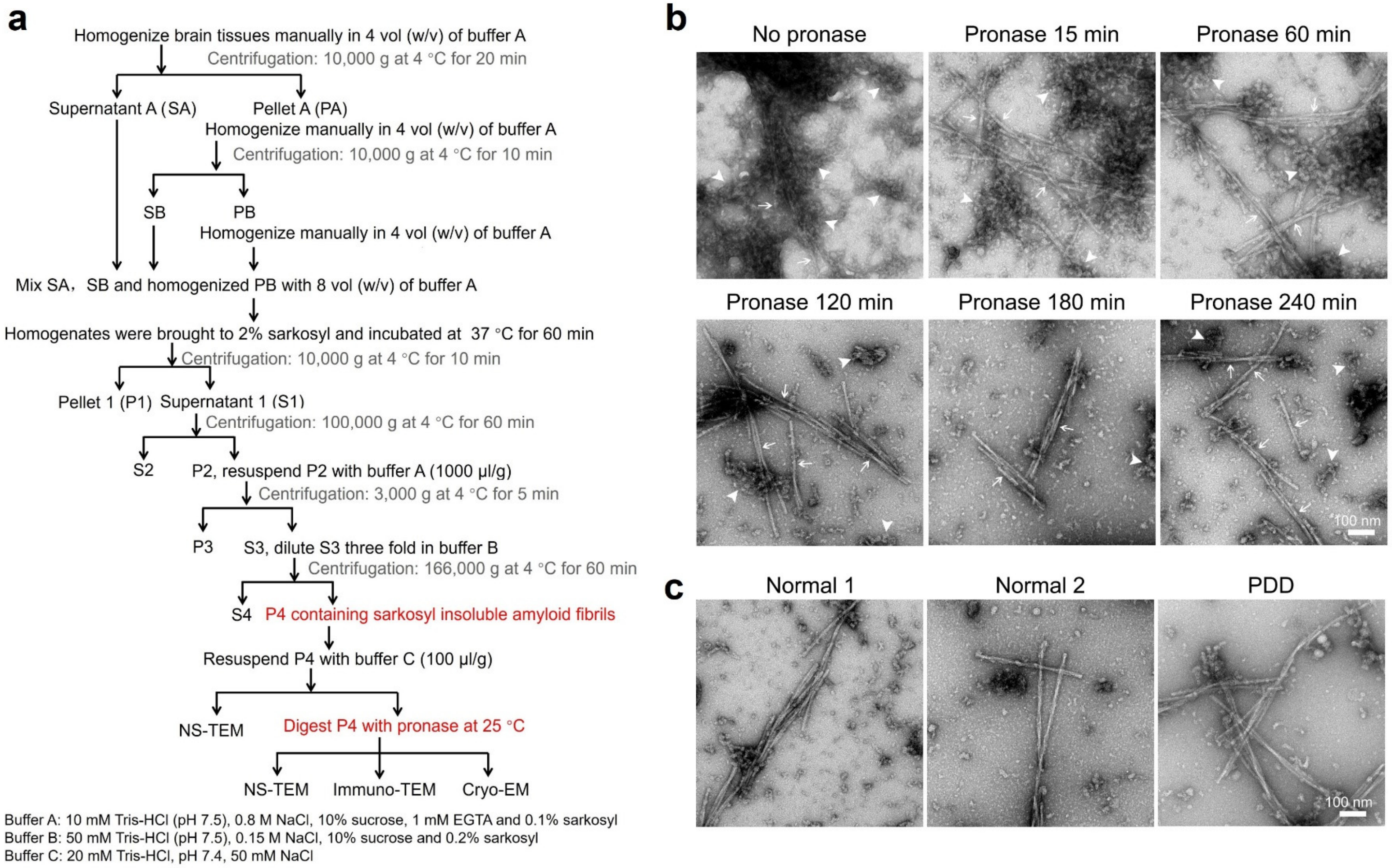

Fig. S2. Extraction of amyloid fibrils from human brain tissues. a. Workflow of sequential extraction of amyloid

fibrils from human brains. After homogenization, incubation with 2% sarkosyl and several times of centrifugation to remove most of soluble proteins and other contaminants such as collagen, pellet 4 (P4) was acquired. P4 is sarkosyl insoluble pellet that contains amyloid fibrils and is incubated with pronase to remove remaining contaminant proteins. **b.** NS-TEM micrographs of P4 extracted from the PDD patient with the treatment of pronase for the indicated period of time. Amyloid fibrils and amorphous aggregates are indicated with white arrows and white arrow heads, respectively. **c**. NS-TEM micrographs of amyloid fibrils extracted from the brains of normals 1, 2 and PDD, respectively.

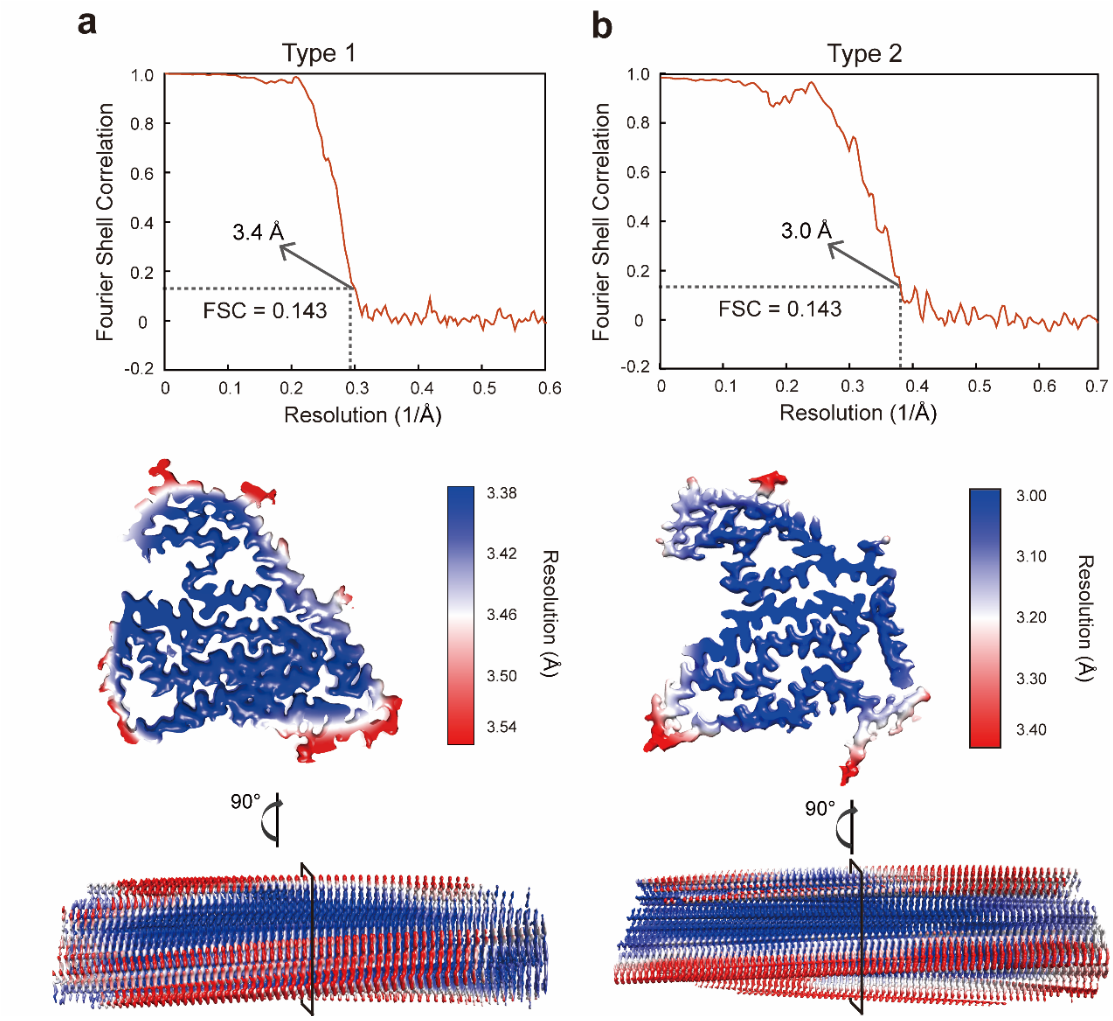

Fig. S3. Local resolution estimations of the cryo-EM density maps of Type 1 and Type 2 fibrils from normal 2 and PDD. Gold-standard Fourier shell correlation (FSC) curves (top) and local resolution estimations (bottom) of density maps of Type 1 (**a**) and Type 2 (**b**) TMEM106B fibrils. The overall resolution of the Type 1 (**a**) and Type 2 (**b**) fibrils is 3.4 Å and 3.0 Å, respectively.

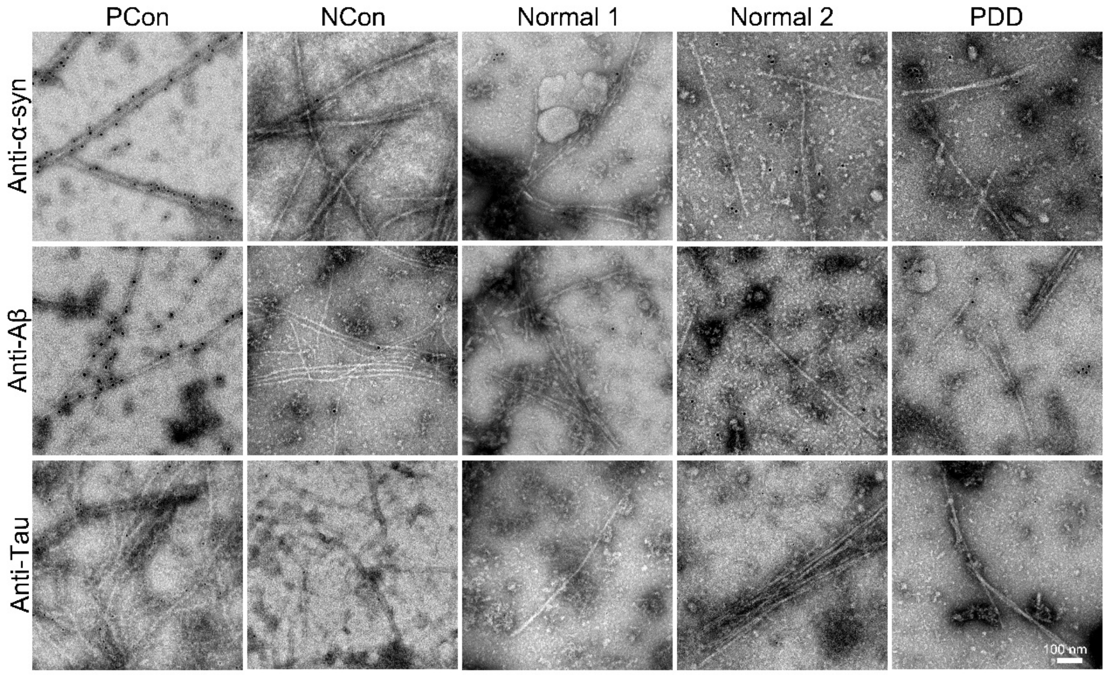

Fig. S4. Representative immuno-gold negative-staining electron microscopic micrographs of brain-extracted amyloid fibrils. The amyloid fibrils extracted from brains of the three donors were incubated with anti-α-syn, anti-tau and anti-Aβ antibodies, respectively. *In vitro* assembled α-syn, tau and Aβ fibrils in the positive control (PCon) group were labeled by immunogold, but not those in the negative control (NCon) group or in the brain-extracted amyloid fibrils. Scale bar, 100 nm.

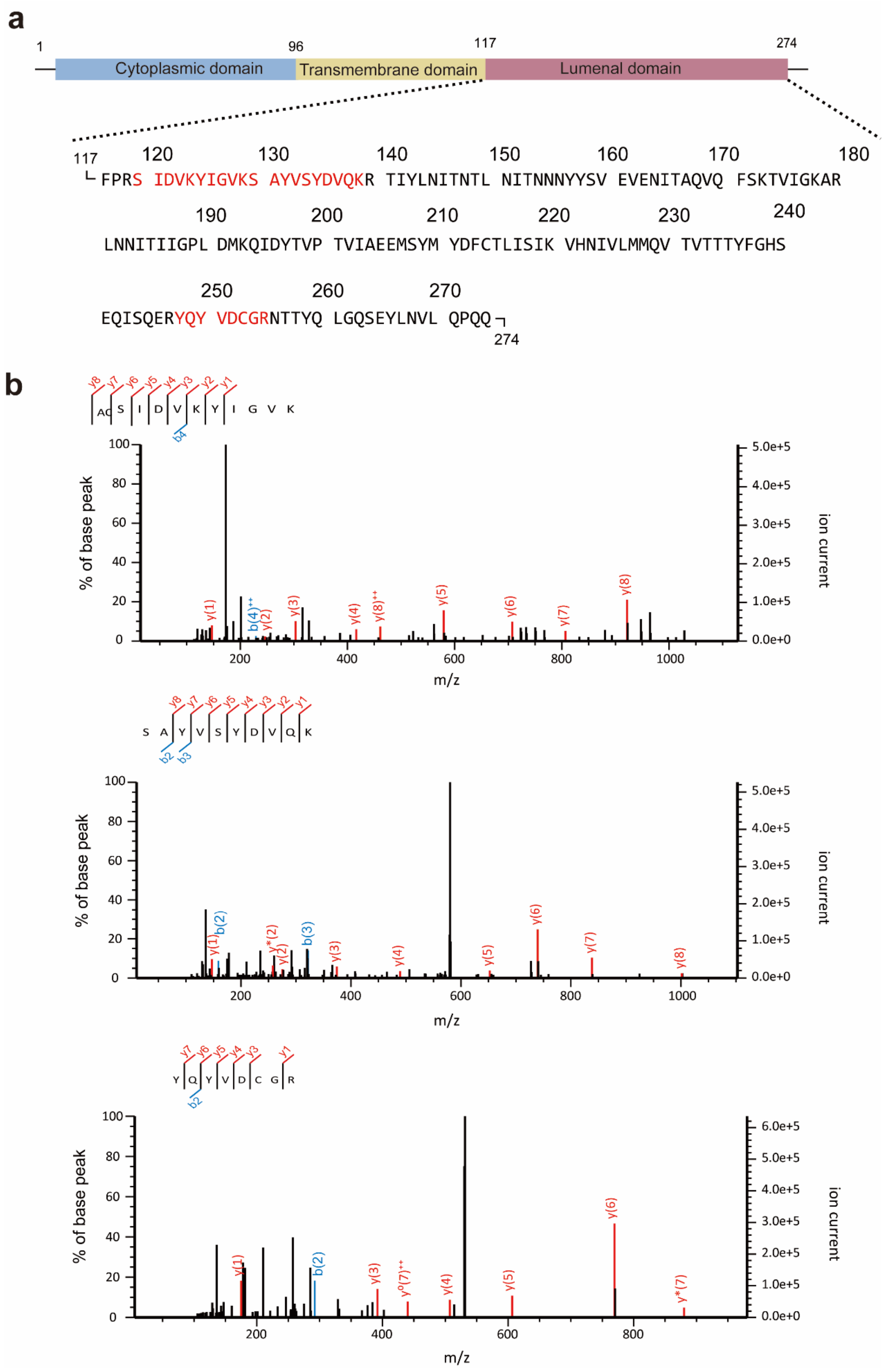

Fig. S5. Identification of TMEM106B fibrils by mass spectrometry. a. TMEM106B contains three domains including the cytoplasmic, the

transmembrane and the luminal domain. The primary sequence of the luminal domain is presented, with the sequences of unique peptides detected by LC-MS/MS highlighted in red. **b.** The MS/MS spectrum of the three detected peptides from P4 sample of PDD, the fragments with b and y ions are labeled in blue and red accordingly.

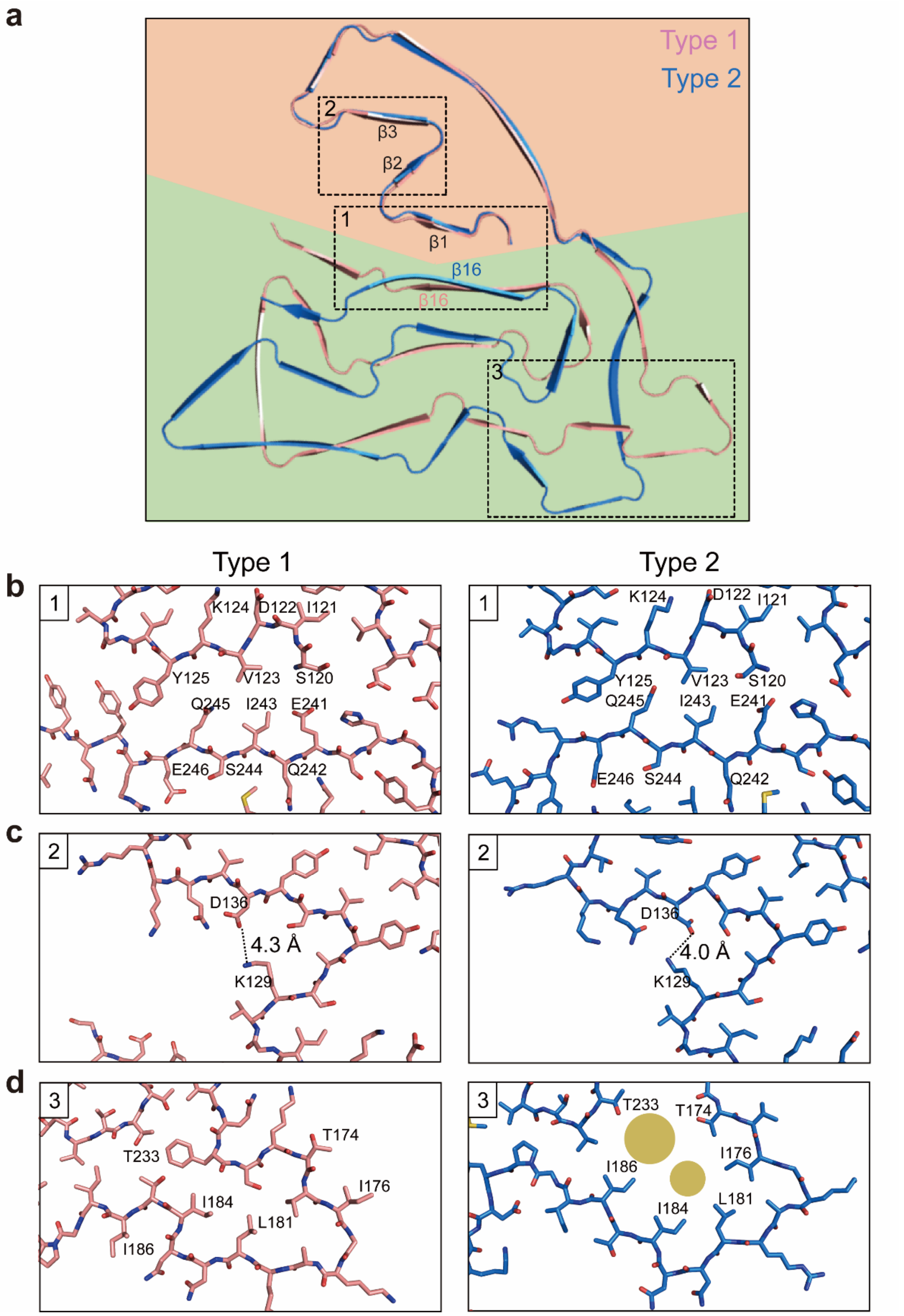

Fig. S6. Structure comparison of Type 1 and Type 2 TMEM106B fibrils. a. The structures of Type 1 and Type 2 fibrils are superimposed. Type 1 is colored in light pink. Type 2 is in sky blue. The background of the two parts of the curling stone-like fold is colored differently: the handle part is in orange, and the stone

part is in green. **b.** Zoom-in views of Box 1 in (**a**) showing the steric-zipper like interaction between β1 and β16. **c.** Zoom-in views of Box 2 in (**a**) showing the salt bridge formed by K129 and D136. The distance between the two residues is indicated. **d.** Zoom-in views of Box 3 in (**a**) showing the two extra densities (yellow dots) in the structure of Type 2 fibril.

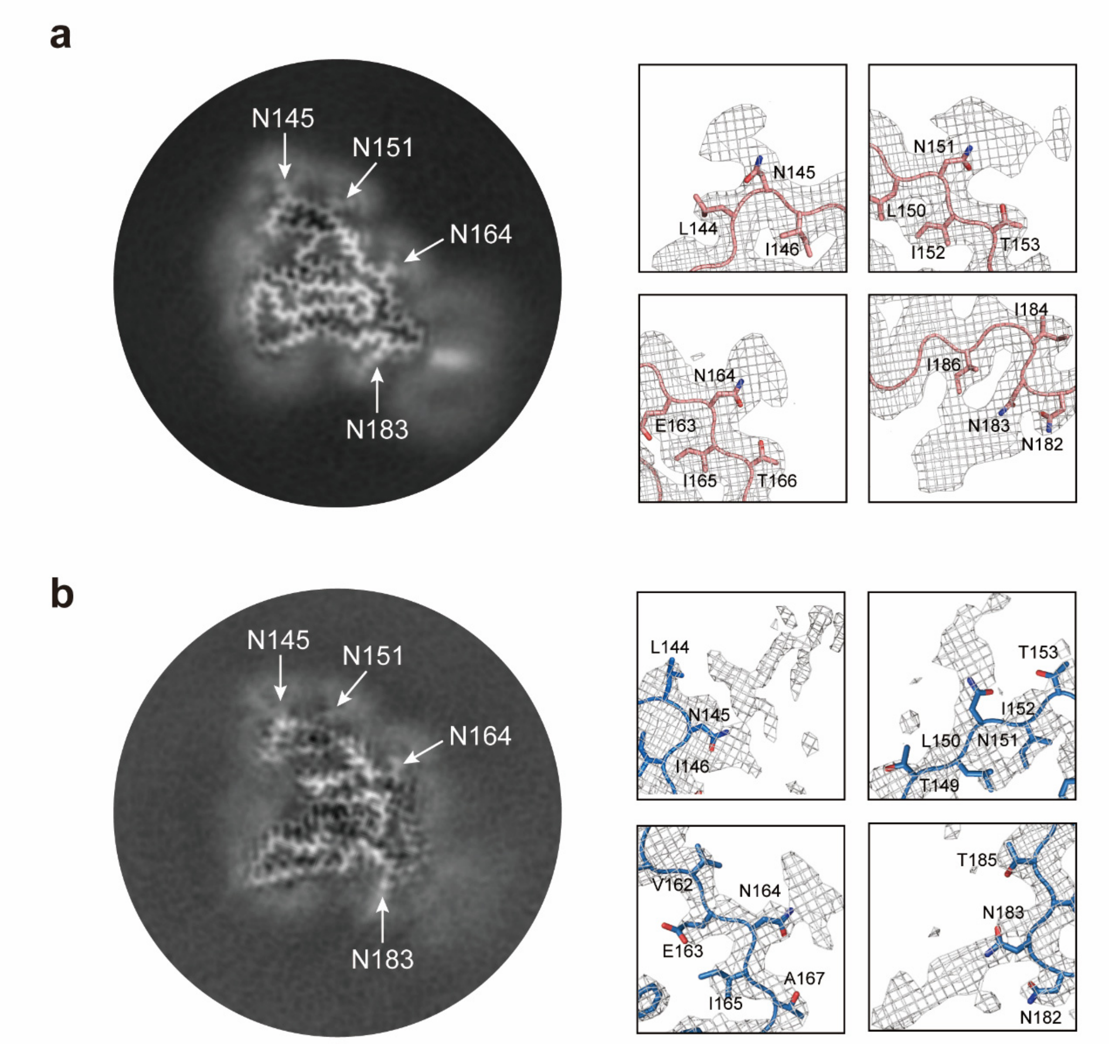

Fig. S7. Extra densities in the density maps of Type 1 (normal 2) and Type 2 (PDD) TMEM106B fibrils. Cross-sections of the 3D density maps of Type 1 (**a**) and Type 2 (**b**) TMEM106B fibrils (left). The four large additional electron densities are indicated by white arrows. Enlarged views of the segments containing the four potentially glycosylated Asn residues of the TMEM106B structural model in the electron density (mesh).

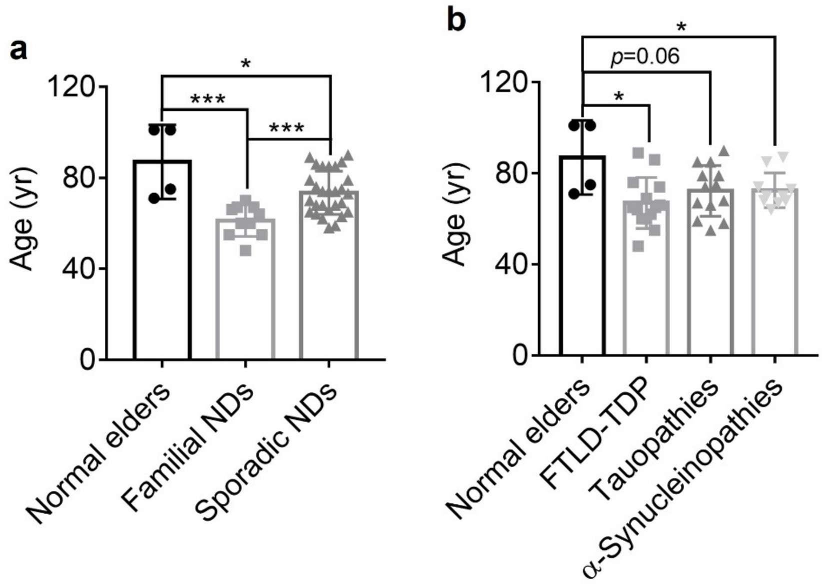

Fig. S8. Comparison of the ages of donors with TMEM106B fibrils. a. Comparison of the ages of normal elders, patients with familial NDs, and patients with sporadic NDs. Normal elders, n=4, age (mean ± SD), 87 ± 16.25; Familial NDs, n=10, age (mean ± SD), 61.2 ± 6.91; Sporadic NDs, n=28, age (mean ± SD), 73.43 ±9.48. **b.** Comparison of the ages of normal elders, patients with FTLD-TDP, tauopathies and α-synucleinopathies. Normal elders, n=4, age (mean ± SD), 87 ± 16.25; FTLD-TDP, n=14, age (mean ± SD), 67.07 ± 11.18; Tauopathies, n=13, age (mean ± SD), 72.31 ±11.19; α-Synucleinopathies, n=10, age (mean ± SD), 72.6 ±7.63. $^{*}p < 0.05$, $^{***}p < 0.001$. Two-tailed unpaired Student’s t-test.

Supplementary Tables

Table S1. Overview of the subjects used in this study.

Case	Sex	AAO (yr)	AAD (yr)	Clinical diagnosis	Cause of death	Pathology	Brain regions
Normal 1	M	NA	71	Non-ND	Not reported	NA	Temporal cortex
Normal 2	M	NA	101	Non-ND	Old age	Aβ and tau aggregates	Temporal cortex
PDD[a]	F	50	70	PDD	Colon cancer with systematic metastasis	α-syn aggregates	Frontal cortex

AAD, age at death; AAO, age at onset; F, female; M, male; ND, neurodegenerative disease; NA, not applicable; PDD, Parkinson's disease with dementia

[a]The PDD patient had a definite family history, but no definite pathogenic gene mutation was detected by whole genome sequencing.

Table S2. Clinical characteristics of the PDD patient in the last visit to hospital.

	PDD
Disease duration (y)	18
H&Y	5
UPDRS-III score	57
NMSS	105
GDS score	16
BDI	23
ESS	13
RBDSQ score	7
SSST-12 score	—[a]
MMSE	12
PDQ-39	82

[a]The post-PD patient could not accomplish the SSST-12 test

H&Y, Hoehn & Yahr; UPDRS-III, Unified Parkinson's Disease Rating Scale part III; NMSS, Non-Motor Symptoms Scale; GDS, Geriatric Depression Rating Scale; BDI, Beck Depression Inventory; ESS, Epworth Sleepiness Score; RBDSQ, Rapid-Eye-Movement Sleep Behavior Disorder Screening Questionnaire; SSST-12, Sniffin' Sticks screening 12 test; MMSE, Mini Mental State Examination; PDQ-39,39-item Parkinson's disease questionnaire

Table S3. Cryo-EM structure determination and statistics of the model building.

Data collection and processing	Normal 1 Type 2	Normal 2 Type 1	PDD Type 2	PDD Type 3
	-	(EMD-33054)	(EMD-33055)	-
	-	(PDB 7X83)	(PDB 7X84)	-
**Data Collection**				
Magnification	105,000	105,000	105,000	105,000
Pixel size (Å)	0.83	0.83	0.83	0.83
Defocus Range (μm)	-1.4 to -2.2	-1.4 to -2.2	-1.4 to -2.2	-1.4 to -2.2
Voltage (kV)	300	300	300	300
Camera	BioContinuum K3	BioContinuum K3	BioContinuum K3	BioContinuum K3
Microscope	Krios G4	Krios G4	Krios G4	Krios G4
Exposure time (s/frame)	0.05	0.05	0.05	0.05
Number of frames	40	40	40	40
Total dose ($e^-/Å^2$)	55	55	55	55
**Reconstruction**				
Micrographs	1,041	989	440	440
Manually picked fibrils	6,856	5,963	1,142	1,142
Box size (pixel)	686	360	440	1,024
Inter-box distance (Å)	57	30	37	85
Initial particle images (no.)	68,554	79,502	25,081	25,081
Final particle images (no.)	7,215	68,607	17,752	3,171
Resolution (Å)	4.9	3.4	3.0	5.1
Map sharpening B-factor ($Å^2$)	-	-128.746	-58.276	-
Helical rise (Å)	-0.42	-0.71	-0.45	-0.42
Helical twist (°)	4.82	4.84	4.83	4.83
**Atomic model**				
Non-hydrogen atoms	-	3,258	3,258	-
Protein residues	-	405	405	-
Ligands	-	0	0	-
r.m.s.d. Bond lengths	-	0.009	0.008	-
r.m.s.d. Bond angles	-	0.821	0.807	-
All-atom clash score	-	16.59	9.22	-
Rotamer outliers	-	0%	0%	-
Ramachandran Outliers	-	0%	0%	-
Ramachandran Allowed	-	15.79%	14.29%	-
Ramachandran Favored	-	84.21%	85.71%	-

Table S4. Summaries of donors with TMEM106B fibrils.

Case	Disease	Age (yr)	Familial or sporadic	Reference
1	AD	79	Sporadic	Schweighauser et al[8]
2	FAD	67	Familial	Schweighauser et al[8]
3	EOAD	58	Sporadic	Schweighauser et al[8]
4	PA	59	Sporadic	Schweighauser et al[8]
5	CBD	74	Sporadic	Schweighauser et al[8]
6	CBD	79	Sporadic	Schweighauser et al[8]
7	FTDP-17T	55	Familial	Schweighauser et al[8]
8	AGD	85	Sporadic	Schweighauser et al[8]
9	AGD	90	Sporadic	Schweighauser et al[8]
10	LNT	66	Sporadic	Schweighauser et al[8]
11	ARTAG	85	Sporadic	Schweighauser et al[8]
12	PD	87	Sporadic	Schweighauser et al[8]
13	PDD	64	Sporadic	Schweighauser et al[8]
14	FPD	67	Familial	Schweighauser et al[8]
15	DLB	74	Sporadic	Schweighauser et al[8]
16	DLB	73	Sporadic	Schweighauser et al[8]
17	MSA	85	Sporadic	Schweighauser et al[8]
18	MSA	70	Sporadic	Schweighauser et al[8]
19	MSA	68	Sporadic	Schweighauser et al[8]
20	FTLD-TDP-A	66	Familial	Schweighauser et al[8]
21	FTLD-TDP-C	65	Sporadic	Schweighauser et al[8]
22	ALS	63	Sporadic	Schweighauser et al[8]
23	Normal control	75	NA	Schweighauser et al[8]
24	Normal control	101	NA	Schweighauser et al[8]
25	FTLD-TDP-A	60	Familial	Chang et al[9]
26	FTLD-TDP-A	55	Familial	Chang et al[9]
27	FTLD-TDP-A	60	Familial	Chang et al[9]
28	FTLD-TDP-A	89	Sporadic	Chang et al[9]
29	FTLD-TDP-A	48	Familial	Chang et al[9]
30	FTLD-TDP-B	62	Sporadic	Chang et al[9]
31	FTLD-TDP-B	74	Sporadic	Chang et al[9]
32	FTLD-TDP-C	69	Sporadic	Chang et al[9]
33	PSP	68	Sporadic	Chang et al[9]
34	PSP	75	Sporadic	Chang et al[9]
35	DLB	68	Sporadic	Chang et al[9]
36	FTLD-TDP-A	86	Sporadic	Jiang et al[10]
37	FTLD-TDP-B	76	Sporadic	Jiang et al[10]
38	FTLD-TDP-C	65	Sporadic	Jiang et al[10]
39	FTLD-TDP-D	64	Familial	Jiang et al[10]
40	PDD	70	Sporadic	This work
41	Normal control	71	NA	This work
42	Normal control	101	NA	This work

AD: sporadic Alzheimer’s disease; AGD: argyrophilic grain disease; ALS: amyotrophic lateral sclerosis; ARTAG: aging-related tau astrogliopathy; CBD: corticobasal degeneration; DLB: dementia with Lewy bodies; EOAD: sporadic early-onset Alzheimer’s disease; FAD: familial Alzheimer’s disease; FPD: familial Parkinson’s disease; FTDP-17T: familial frontotemporal dementia and parkinsonism linked to chromosome 17 caused by *MAPT* mutations; FTLD-TDP-A: frontotemporal lobar degeneration with TDP-43 inclusions type A; FTLD-TDP-B: frontotemporal lobar degeneration with TDP-43 inclusions type B; FTLD-TDP-C: frontotemporal lobar degeneration with TDP-43 inclusions type C; FTLD-TDP-D: frontotemporal lobar degeneration with TDP-43 inclusions type D; LNT: limbic-predominant neuronal inclusion body 4R tauopathy; MSA: multiple system atrophy; NA: not appliable; PA: pathological aging; PART: primary age-related tauopathy; PD: sporadic Parkinson’s disease; PDD: Parkinson’s disease dementia; PSP: progressive supranuclear palsy; VaD: vascular dementia.

Supplementary references

1 Schweighauser M, Shi Y, Tarutani A *et al.* Structures of α-synuclein filaments from multiple system atrophy. *Nature* 2020; **585**:464-469.

2 Yang Y, Arseni D, Zhang W *et al.* Cryo-EM structures of amyloid-β 42 filaments from human brains. *Science (New York, NY)* 2022; **375**:167-172.

3 Zheng SQ, Palovcak E, Armache JP, Verba KA, Cheng Y, Agard DA. MotionCor2: anisotropic correction of beam-induced motion for improved cryo-electron microscopy. *Nature methods* 2017; **14**:331-332.

4 Rohou A, Grigorieff N. CTFFIND4: Fast and accurate defocus estimation from electron micrographs. *Journal of structural biology* 2015; **192**:216-221.

5 Scheres SHW. Amyloid structure determination in RELION-3.1. *Acta crystallographica Section D, Structural biology* 2020; **76**:94-101.

6 Emsley P, Lohkamp B, Scott WG, Cowtan K. Features and development of Coot. *Acta crystallographica Section D, Biological crystallography* 2010; **66**:486-501.

7 Adams PD, Afonine PV, Bunkóczi G *et al.* PHENIX: a comprehensive Python-based system for macromolecular structure solution. *Acta crystallographica Section D, Biological crystallography* 2010; **66**:213-221.

8 Schweighauser M, Arseni D, Huang M *et al.* Age-Dependent Formation of TMEM106B Amyloid Filaments in Human Brain. *BioRxiv* 2021:2021.2011.2009.467923. (published online in *Nature* 2022)

9 Chang A, Xiang X, Wang J *et al.* Homotypic fibrillization of TMEM106B across diverse neurodegenerative diseases. *Cell* 2022.

10 Jiang YX, Cao Q, Sawaya MR *et al.* Amyloid fibrils in frontotemporal lobar degeneration with TDP-43 inclusions are composed of TMEM106B, rather than TDP-43. *BioRxiv* 2022:2022.2001.2031.478523. (published online in *Nature* 2022)
